# Mutations Change the Boolean Logic of Gene Regulation

**DOI:** 10.1371/journal.pbio.0040064

**Published:** 2006-03-28

**Authors:** Richard Robinson

It is easy to think of a gene acting like a light bulb, switching either on or off, remaining silent, or being transcribed by the RNA-making machinery. The region of DNA that controls the gene's output is called its regulatory region, and in this simple (and too simplistic) scenario, that region would act like a simple on–off switch.

But the regulatory regions of real genes are more complex, and act more like molecular computers, combining the effects of multiple inputs and calibrating the gene's output accordingly. The inputs are the various molecules that affect gene activity by binding to sites in the regulatory region. These molecules combine their effects in complex ways. Sometimes the gene remains silent unless both are present. Sometimes they are additive, such that the output when two factors are present is twice the output when only one is present. Sometimes they cancel each other out—in the presence of either, the gene is transcribed, but in the presence of both, it is not.

Thus, the regulatory region acts as a Boolean logic function, whose simple ANDs, ORs, and NOTs combine to determine the output of the gene. In a new study, Avi Mayo, Uri Alon, and colleagues show that mutations in the regulatory region affect this logic function in a simple and well-studied genetic system, the *lac* operon in *Escherichia coli* bacteria, whose suite of genes regulate metabolism of lactose.

The authors began by creating multiple strains of bacteria with mutations in the binding sites for the two regulators of the gene, called CRP and LacI, that respond to cyclic AMP and IPTG, an analog of lactose. They analyzed the effect of these mutations on the rate of gene transcription in the presence of varying concentrations of the two inducers. Previously, the authors showed that the function of the unmutated regulatory region was intermediate between a pure “AND gate” (in Boolean parlance) and a pure “OR gate”: that is, at certain concentrations the first regulator AND the second were needed, but at others, one OR the other sufficed. In the mutated strains, they found that some mutations replicated this behavior, while others switched the regulatory region to a more purely AND or purely OR gate, independent of concentration. Some mutations left the regulator almost like a simple light switch, whose on-or-off state depended almost entirely on one, but not the other, regulator.

Next, they developed a mathematical model that links the binding strengths of the regulators for each mutation (the “inputs” of the “regulatory function”) to the gene output. Based on this model, they propose that point mutations in this system cannot create all of the 16 possible two-input gates described by Boolean logic. For instance, since both regulators stimulate gene activity, no simple mutation is likely to switch the system to an “AND NOT” gate, in which one input can stimulate only when the other is not present.

The authors suggest that applying this kind of logic analysis to genetic “circuits” may aid in the design of artificial genetic systems, and in understanding more complex gene regulatory regions. With only 30,000 genes, it is clear that humans and other complex creatures must depend on exquisitely regulated gene expression to develop and adapt to environmental changes. The findings in this study support the growing appreciation that, from bacterium to baleen whale, complexity is highly dependent on fine-tuning gene regulation.

## 

**Figure:**
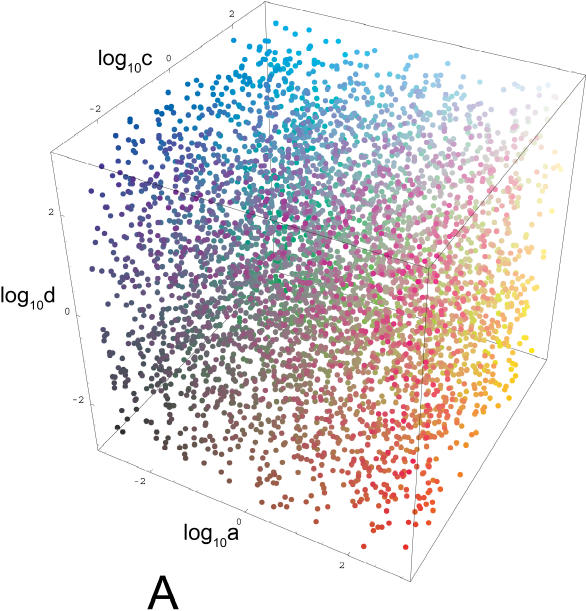
Each point on this graph represents three parameters that describe the binding affinities of RNAP, Lacl, and CRP to the *lac* operon.

